# Sensory Insights in Aging: Exploring the Impact on Improving Dietary Through Sensory Enhancement

**DOI:** 10.1002/fsn3.70074

**Published:** 2025-03-03

**Authors:** Yilin Li, Shuying Wang, Lanxin Zhang, Qianhui Dong, Xinyu Hu, Yuxin Yang, Ting Liu, Baopei Wu, Bingqi Shan, Chuncao Yin, Qinggang Xie, Baoqing Zhu, Chengdong Zheng

**Affiliations:** ^1^ Heilongjiang Feihe Dairy Co., Ltd Beijing China; ^2^ Beijing Key Laboratory of Food Processing and Safety in Forestry, Department of Food Science, College of Biological Sciences and Biotechnology Beijing Forestry University Beijing China; ^3^ Department of Psychology, School of Humanities and Social Science Beijing Forestry University Beijing China

**Keywords:** aging population, dietary behavior, elderly nutrition, food product design, sensory evaluation, sensory impairment

## Abstract

The trend of global population aging is becoming increasingly evident, with the proportion of the elderly population continuously rising, making it one of the most profound demographic trends of the 21st century. As people age, their sensory functions generally decline, such as vision, hearing, smell, and taste, which not only affects their food choices and enjoyment but can also lead to health issues like malnutrition and weight loss. In addition, chronic diseases such as diabetes, cardiovascular diseases, and the COVID‐19 pandemic can exacerbate sensory impairments in older adults. Currently, most sensory evaluation methods are designed for healthy adults and have limitations when applied to the elderly, such as visual impairment making it difficult to see scoring sheets or linear scales, and hearing impairment preventing understanding of questions and requests from sensory analysts, leading to potential biases and inaccuracies in data collection. Therefore, there is an urgent need to develop sensory evaluation systems suitable for older adults to better understand and address their sensory changes due to aging and health conditions. This review summarizes the sensory abilities, cognitive functions, and physical health status of older adults; explores how to improve their food intake and appetite through flavor‐enhanced foods; and reviews current sensory evaluation methods, pointing out their limitations and the necessity for developing new approaches to meet the diverse needs of older adults. Future research should deepen the understanding of the neurobiological mechanisms of sensory decline, develop implicit sensory evaluation methods based on EEG and facial microexpressions, and optimize personalized sensory compensation strategies that are safe and sustainable in order to improve the dietary health and quality of life of older adults.

## Graphical abstract

Aging impacts sensory abilities in older adults. Sensory impairment in older people displays different degrees and aspects. Food sensory compensation strategies have different effects on food intake.

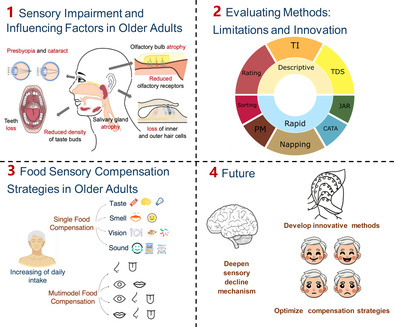

## 1 Introduction

The proportion of elderly people in the world's population is increasing, and population aging may become one of the most dominant social trends in the 21st century. According to the World Population Prospects 2022, the proportion of people aged 65 and over will be 10% globally in 2023 and is projected to reach 16.5% by 2050, which means that one in six people will be aged 65 and over (United Nations 2022). In 2022, 19.8% of China's population was aged 60, with 14.9% of the population aged 65 and over (National Bureau of Statistics of China 2020). It is expected that in 2035, China's elderly population will exceed 400 million (NHSC 2022).

Today, most of the products in the market are aimed at young people, and as the number of elderly people increases, there is a need to give more consideration to their needs and designing food products according to their own characteristics. Many studies have confirmed that as people age, their senses deteriorate to varying degrees and that sensory changes can have an impact on food selection and enjoyment (Davenport 2004). Impairments in sensory perception are often associated with aging. Visual and auditory impairments may be widely recognized as sensory impairments, followed by perceptual impairments such as taste, smell, and texture (Doty and Kamath 2014; Hutchings et al. 2014; Withers et al. 2013). Older adults also often face other physical sensory impairments such as dysphagia, wearing dentures, or dysfunction. They may also be taking medication and may have psychosensory impairments such as diminished cognition and poor mental performance, which may reduce their ability to perceive sensory stimulation and may lead to insufficient food intake, placing them at risk for protein deficiency and undernutrition (Doets and Kremer 2016). Older adults have a growing need for variety and richness in the products they consume, and understanding these needs and how they change is a key step in designing products for older adults. Our work summarizes the deficits in sensory functions (taste, smell, vision, hearing, chemosensation) and their causes, and the effects of COVID‐19 on sensory perception (mainly focusing on taste and smell) in older adults.

Sensory evaluation is the process of evaluating food in order to accurately measure human responses to it and to minimize potential nonsensory influences such as brand effects (Lawless and Heymann 2010). This work provides an overview of sensory evaluation methods currently used with older adults. These methods include not only difference tests, descriptive analysis tests, and ranking tests but also experiments measuring the emotions of older adults, such as preference, familiarity, and acceptability. Meiselman argues that dietary behaviors consist of three variables: the food, the person, and the environment (which includes all environmental factors; the physical environment in which the shopping and eating takes place, as well as the social and economic context) (Meiselman 2007). Currently, most sensory methods are conducted with “healthy” consumers as test subjects. The applicability of these methods to healthy or frail older adults has yet to be fully considered, and some researchers have not focused on older adults. However, in today's society, where there is a large elderly population, it is increasingly important to develop sensory evaluation systems that are appropriate for them and then to produce tasty and nutritious food for them. Some of the existing sensory testing methods may not be friendly to the elderly with sensory impairments; for example, the visually impaired are unable to see the scoring sheet or line scale; the hearing impaired are unable to understand the questions and requests from the sensory analyst, and have poor dexterity in tasting or categorizing the samples and have difficulty in writing and expressing themselves; and subjects may easily become fatigued if the sample size set is too large. All these factors can impose restrictions on the application of sensory evaluation methods in various ways. It is crucial to consider these aspects when designing sensory studies involving older consumers. However, aside from a few exceptions (Duffy et al. 1999; Griep et al. 1997; Mojet, Christ‐Hazelhof, et al. 2005, Mojet, Köster, et al. 2005), the majority of sensory studies overlook the specificity of older adults.

This work categorizes older adults, encompassing both “healthy” and “frail” older adults, as well as those living independently and residing in nursing homes. Older adults living in nursing homes, as well as those who are frail, are mentioned. Additionally, both older persons living independently and those residing in nursing homes are discussed. These groups of older adults differ considerably in terms of their sensory abilities (sight, hearing, taste, smell, and mouth), cognitive abilities, and physical health. The effects of flavor‐enhancing foods on food intake, appetite, preference, and acceptability in older adults and measures to increase food intake in older adults are now more widely accepted.

We conducted a systematic review of the sensory function deficits in older adults and their causes, as well as the methods used for sensory evaluation, food preference, and intake assessment in this population. Furthermore, we explored the need for and measures of increasing food intake in older adults through enhancing sensory attributes. Building on this foundation, we summarized the current research and proposed new ideas and perspectives.

## 2 Sensory Impairment and Influencing Factors in Older Adults

Food enjoyment is a key aspect of the human appetite and affects psychological and physical health, relationships, and self‐esteem, potentially leading to long‐term implications for recovery, especially for older adults (Drewnowski and Evans 2001; Lumbers and Raats 2006; Abrahams 2020). Age‐related changes in sensory perception have become a consensus. Sensory impairment in older adults displays different degrees and aspects. In fact, the phenomenon of impairment of sensory perception in older adults is associated with different reasons, such as physical state, psychological state, and others (Table 1). The impairment of oral function for food texture perception has been reviewed very recently by Liu et al. (2022). This section will examine the sensory perception state (especially, taste and smell) of older adults and the main causes of related impairment.

**TABLE 1:** Examples of sensory impairment in the elderly.

Information reference	Participants' information	Main finding
Laska (2001)	Age: 100 participants aged 65–88 years Area: Germany	The ability to discriminate chemical stimuli with trigeminal properties decreases with aging in the elderly
Hummel et al. (2003)	Age: 35 patients with olfactory Dysfunction aged 28–69 years; 17 normosmic subjects aged 28–82 years Area: Germany	The sensitivity of the intranasal trigeminal nerve decreases in older adults as they age
Mojet (2003)	Age: 21 participants aged 60–75 years Area: Wageningen	Perception of salty taste decreases with age in the elderly
Koskinen and Tuorila (2005)	Age: 27 participants aged 66–83 years Area: Helsinki	The elderly have some difficulty in distinguishing the intensity of odors
Fukunaga et al. (2005)	Age: 30 participants aged 65–85 years Area: Japan	Compared to young people, older adults have higher thresholds with the four basic taste sensations
Kremer et al. (2007b)	Age: 22 participants aged 60–85 years Area: the Netherlands	Decreased olfactory sensitivity in the elderly affects their evaluation of taste intensity
Baraldi et al. (2007)	Age: 211 participants aged 60–99 years Area: Brazil	Hearing loss deepens with age and is greater in the 80–89‐year‐old age group with higher frequency damage
Kawagishi et al. (2009)	Age: 60 participants aged 66–91 years Area: Japan	Compared to young people, the ability to recognize the tongue in three dimensions is significantly reduced in the elderly
Solemdal et al. (2012a, 2012b)	Age: 174 participants aged 70–103 years Area: Germany	Oral diseases can affect the perception of sweet and salty tastes in the elderly
Sasano et al. (2015)	Age: 82 participants aged 65–89 years Area: Japan	Aging causes the elderly to lose their sense of taste, but four basic taste sensations remain
Sulmont‐Rosse et al. (2015)	Age: 559 participants aged 65–99 years Area: France	Aging can lead to impairment of chemosensory abilities in the elderly
Yoshinaka et al. (2015)	Age: 949 participants aged 79–81 years Area: Japan	Loss of taste sensation in the elderly with aging
Joussain et al. (2015)	Age: 32 anosmic participants aged 30–72 years; 32 hyposmic participants aged 27–75 years; 32 controls participants aged 28–73 years Area: Lyon, Geneva, Helsinki	The prevalence of olfactory dysfunction in people over 60 years (39%) are significant higher than people between 18 and 28 years (10%)
Piotrowicz et al. (2016)	Age: 3471 participants aged 65–104 years, mean age 78.3 years Area: Poland	Vision impairment, hearing impairment, and cognitive impairment increased with age
Cui et al. (2017)	Age: 388 participants aged 50–59 years; 413 participants aged 60–69 years; 157 participants aged 70–79 years; 47 participants aged 80 years Area: China	Vision impairment mainly occurred in Chinese participants aged 70–80 years and increases as the aging process progresses
Chen et al. (2017)	Age: 2975 participants aged 60 years Area: 1818 participants were non‐Hispanic White	Declining cognitive function in the elderly can lead to distance vision impairment
Birte‐Antina et al. (2017)	Age: 91 participants aged 50 to 84 years Area: Germany	As the elderly age, the sense of smell gradually loses its function
Cavazzana et al. (2018)	Age: 104 participants aged 50–100 years Area: Norway	Hearing threshold changes significantly with age
Mazzatenta et al. (2020)	Age: 100 participants aged 28–94 years Area: United States	COVID‐19 patients experience hyposmia and loss of smell, but no relationship was found between the severity of olfactory impairment and age
Manesse et al. (2021)	Age: 510 participants aged 50–59 years; 202 participants aged 60–89 years Area: France	The prevalence of olfactory disorders is high in the French population and is more prevalent in the elderly population
Chaaban et al. (2021)	Age: 102 participants aged 19–69 years Area: Denmark	COVID‐19 causes loss of the senses of smell and taste.
Yang et al. (2022)	Age: 24 participants aged 61–73 years Area: China	Aging significantly reduced gustatory sensitivity (sweetness) but not chemical anesthesia sensitivity in older adults

### 2.1 Impact of Aging on Sensory Impairment in Older Adults

**FIGURE 1:**
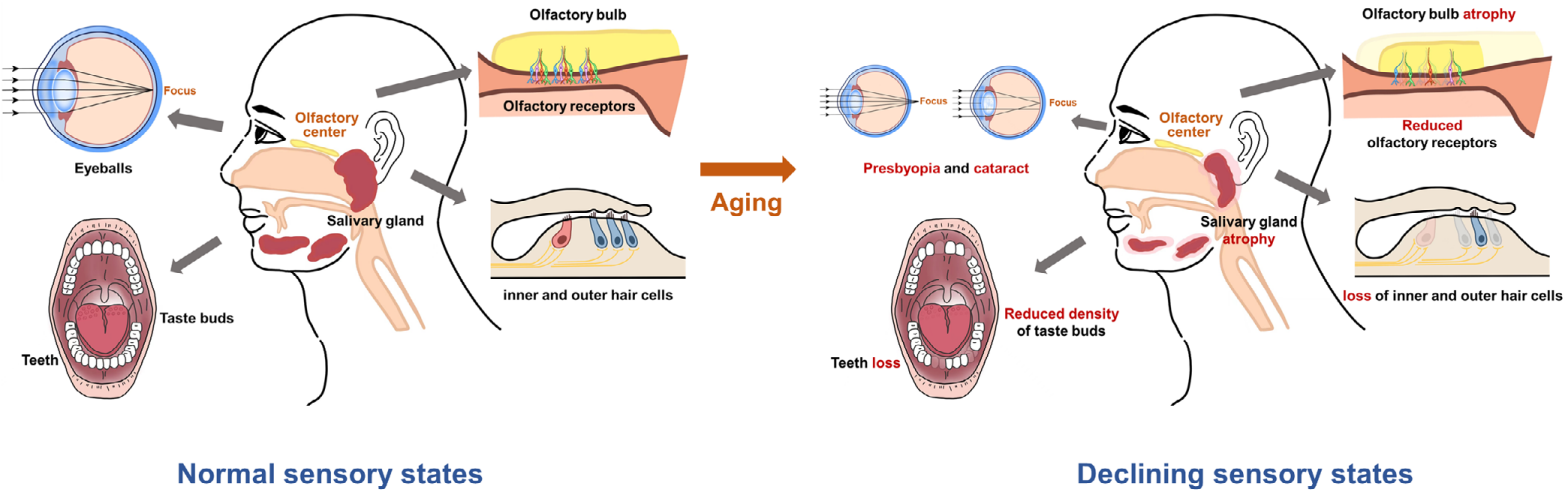
Schematic diagram of sensory decline.

Numerous studies on older adults have found that a person's physiological status is associated with their quality of life (Ahmed and Haboubi 2010; Doets and Kremer 2016; Crimmins 2017). In terms of sensory in people's physiological status, the formation of sensory perception cannot be achieved without the functioning of the nervous system. Sensory organs receive stimuli and convert them into nerve impulses, which are transmitted through afferent nerves to the cortical sensory centers of the brain, thus producing sensation (Watson et al. 2010; Connor et al. 2018). Throughout the natural progression from adolescence to adulthood and to old age, the nervous system undergoes various changes that can impair sensory functions, potentially leading to a decline in olfaction, taste, hearing, vision, and chemosensory perception in older adults (Figure 1).

#### 2.1.1 Visual Impairment in Older Adults

Most of the information from the outside world is transmitted to the brain through the visual system. Visual impairment becomes more prevalent with age. Around the age of 40, changes in vision begin to appear (Pathai et al. 2013). Visual impairment in daily life may manifest as malfunctions in the optical system, which are preliminary indicators of the onset of disease or aging, such as optical aberrations and intraocular light scatter of the eye (Artal et al. 2002). The crystalline lens is an active optical element capable of changing the intensity of light throughout the eye by altering its shape, allowing the eye to focus on objects at different distances (Artal 2014). Aging can cause changes in the optical system of the eyes, leading to various visual impairments in the elderly. During the natural aging process, the shape, radii of curvature, dimensions, surface curvatures, and refractive index of the crystalline lens are continuously changing, thus altering the aberrations of the lens (Artal et al. 2002; Berrio et al. 2010; Dubbelman et al. 2001). These physiological changes in the eyes can affect visual image formation in older adults, thereby affecting their daily activities, such as reading, eating, sports, and recognition capabilities.

Vision loss in older adults has been widely documented in numerous studies. Piotrowicz et al. (2016) reported that the prevalence of clinical conditions such as vision impairment (affecting 42% of 3471 Polish participants with a mean age of 78.3 years), hearing impairment, and cognitive impairment increases with age. Similarly, Cui et al. (2017) found that vision impairment predominantly occurs in Chinese individuals aged 70–80 years and becomes more prevalent as people age. Their study involved 388 participants aged 50–59 years, 413 aged 60–69 years, 157 aged 70–79 years, and 47 aged 80 years in China. They noted that the severity and frequency of vision loss varied significantly by disease, with refractive errors and cataracts being more common, while glaucoma and diabetic retinopathy were less so. Nonrefractive eye diseases, particularly cataracts, also increased in incidence with age. Humes and Young (2016) reported similar findings. Cognitive dysfunction is more common in older adults with visual impairment. Jefferis et al. (2012) found that poor visual acuity is associated with poor performance on the MMSE visual items. Several studies have indicated that visual impairment is a significant factor influencing cognitive decline in older adults (Chen et al. 2017; Cavazzana et al. 2018; Nagarajan et al. 2022).

#### 2.1.2 Hearing Impairment in Older Adults

Hearing impairment can reduce the perceived crispness or crunchiness of food, diminishing the sensation of freshness (Tam and Koppel 2021). The primary mechanism of hearing loss (HL) involves damage to the white matter tracts and gray matter in the cranial region. Specifically, the white matter tracts connect the eighth cranial nerve to subcortical nuclei (e.g., cochlear), while the gray matter is linked to the primary auditory cortices (e.g., Heschl's gyri) (Manno et al. 2021; Feng et al. 2018). Moreover, the effects of HL on the brain are multifocal, impacting all age groups, including adolescents, adults, and older adults (Manno et al. 2021). The auditory system relies on hair cells, sensory cells located on the basement membrane (Purves et al. 2004). Age‐related hearing impairment is primarily caused by degenerative changes in the inner ear structures, such as the loss of inner and outer hair cells, deterioration of spiral ganglion cells, and atrophy of the stria vascularis (Zampini and Spence 2004).

Compared to younger individuals, older adults experience a gradual decline in cognitive ability and speech perception, particularly in noisy or complex auditory environments, due to central auditory dysfunction. This decline is a significant factor in hearing impairment among the elderly (Rutherford et al. 2018; Lin et al. 2013). Neuroimaging studies of age‐related HL have shown that neural activation in response to auditory stimuli decreases with age and neural responses to sound gradually become less sensitive, leading to HL (Peelle et al. 2011; Sheline et al. 2009).

Gerontology accepts the idea that HL is a basic principle associated with aging. Studies have shown that age is one of the most significant factors in the prevalence of sensory impairment, particularly among older adults (Besser et al. 2018; Amieva and Ouvrard 2020; Ellis et al. 2021). Age‐related HL is similar to the cumulative effects of ototoxic substances, noise exposure, diseases, and medical treatments. Typical symptoms of HL include a decline in hearing sensitivity, difficulty interpreting speech in noisy environments, slower processing of central acoustic stimuli, and challenges in sound localization (Hill et al. 2017; Huang et al. 2023). According to Haile et al. (2021), substantial HL affects approximately 62.1% of seniors aged 60–64 years worldwide. With an aging population, this trend cannot be underestimated.

#### 2.1.3 Olfactory Impairment in Older Adults

The olfactory system is stimulated by volatile food molecules, which enhance intake and pleasure and aid in food identification (Tan et al. 2018; Arikawa et al. 2020). The peri‐amygdala and pre‐parietal areas of the cerebral cortex, known as the primary olfactory cortex, are connected to the secondary olfactory cortex. Together, these areas are collectively responsible for the perception of smell (Watson et al. 2010; Wilson and East 2020). Olfactory receptor cells detect odors and transmit signals through nerves to the olfactory bulb, which processes the signals and transmits them to the brain (Cerf Ducastel and Murphy 2003; Connor et al. 2018). The neurological and anatomical morphology of the olfactory system undergoes various changes with age, such as decreased vascularization, fewer olfactory receptors, and olfactory bulb shrinkage, as well as reduced olfactory epithelium thickness and mucus secretion. Additionally, aging may reduce the activity of brain areas that process olfactory signals (Rouby et al. 2002; Manesse et al. 2021). The loss of olfactory neuron selectivity and decreased receptor selectivity to olfactory stimuli can also affect olfactory performance (Rawson et al. 2012; Doty and Kamath 2014; Manesse et al. 2021).

Olfactory function impacts the quality of life, enhances pleasantness and intake, and aids in food recognition (Croy et al. 2014; Manesse et al. 2017). Several studies have indicated that aging is primarily responsible for the decline in olfactory function (Annear et al. 2016; Doets and Kremer 2016; Manesse et al. 2021). According to large‐scale research of the French population by Manesse et al. (2021), they recruited 510 older adults aged 50–59 years and 202 aged 60–89 years. They found that the prevalence of olfactory dysfunction is > 30% in people aged over 60 years, compared to only 10% in those aged 4–12 years. Joussain et al. (2015) also found that the prevalence of olfactory dysfunction in people aged 18–28 years (10%) is significantly lower than in those over 60 years (39%). Additionally, several studies have noted that some older adults' olfactory function is similar to that of younger people, while others have minor deficiencies and some have substantial anosmia (Hummel et al. 2017; Manesse et al. 2021). For instance, Koskinen and Tuorila (2005) found that some elderly people showed similar levels to their younger counterparts in smell tests, while those with low ratings tended to have difficulty differentiating various scent intensities.

#### 2.1.4 Taste Impairment in Older Adults

Taste can be fundamentally divided into five basic types: sweet, sour, bitter, salty, and umami (Gravina et al. 2013). The taste system perceives mainly water‐soluble molecules of taste‐presenting substances. Once these molecules enter the mouth, they dissolve in the saliva and interact with the taste receptors on the tongue (Purves et al. 2004).

Many physiological functions related to taste decline with aging, particularly in the oral cavity. Various types of papillae are distributed on the tongue, such as fungiform, foliate, and circumvallate papillae, with the fungiform papillae mainly located at the front of the tongue and surrounded by taste buds (Srur et al. 2010; Nordin 2017). As the density of taste buds decreases with age, these papillae can also be affected, leading to a decrease in taste perception in older adults (Nordin et al. 2006). Additionally, the oral mucosa becomes thinner with aging, with reduced keratinization and thinning of the epithelial structure (Löe and Karring 1971). The salivary glands, which secrete saliva and facilitate the swallowing of food, gradually atrophy, affecting taste perception in the elderly (Sasano et al. 2015; Imoscopi et al. 2012; Moreira et al. 2006). Dental integrity is also an important factor affecting chewing in the elderly, as tooth loss can lead to the use of dentures, which can impair chewing and oral movements (Kremer, Mojet et al. 2007; Wayler et al. 1984; Sergi et al. 2016).

In fact, the decline in taste perception among older adults is mainly characterized by reduced taste sensitivity and decreased perception intensity. As reported by Murphy and Gilmore (1989), elderly individuals need a 74% increase in caffeine concentration to detect a change, while young individuals only require a 34% increase. This phenomenon occurs only with medium and high concentrations of caffeine, not with low concentrations. According to Yoshinaka et al. (2015), there are age‐related differences in taste sensitivity for the four basic tastes among young and old Japanese, with the sensitivity to sweet taste being more robust than the others. Aging was also found to significantly reduce taste sensitivity to sweetness in the experiments of Yang et al. (2022).

However, the extent of age's influence on detection thresholds varies significantly depending on the specific substances examined (Field and Duizer 2016). Methven et al. (2012) analyzed 69 studies on the impact of aging on taste perception. Some studies showed that the average elevations in detection thresholds were 1.4 times for sweet flavors, 1.5 times for sour flavors, 2.0 times for salty flavors, 2.2 times for bitter flavors, and 2.2 times for umami flavors. They concluded that aging causes a reduction in taste perception for all five basic tastes. The rate of degradation of taste perception with aging varies across different studies. Murphy and Gilmore (1989) found a more substantial impact on sweet taste thresholds with age compared to, for example, bitter taste thresholds. Sasano et al. (2015) found that some older individuals only experience the loss of umami taste sensation while still experiencing the other four basic tastes (sweet, salty, sour, and bitter).

#### 2.1.5 Chemosensory Impairment in Older Adults

The trigeminal nerve primarily mediates chemesthetic sensations, such as stinging (e.g., from carbon dioxide in soda), burning (e.g., chili pepper), cooling (e.g., peppermint), or astringency (e.g., wine) (Nordin 2017). The decline in chemesthetic sensation with increasing age is influenced by multiple factors (Stamps 2019; Oleszkiewicz et al. 2020). Some research suggests that age‐related dysfunction is due to changes in the peripheral olfactory system, including a decrease in olfactory receptor neurons, impaired regeneration capacity, and hindered bacterial clearance (Doty and Kamath 2014; Doty 2018; Claus et al. 2022). Laska's (2001) study showed a significant decrease in older adults' ability to discriminate between chemical stimuli with trigeminal properties compared to younger adults. Hummel et al. (2003) found that older subjects had lower intranasal trigeminal sensitivity than younger subjects. The interaction between the intranasal trigeminal and olfactory systems may lead to a marked decline in trigeminal sensitivity. Kremer et al. (2007a, 2007b) and Kremer, Mojet, et al. (2007) also found that younger individuals performed better in trigeminal sensitivity tests, particularly in detecting menthol and ammonia.

However, Yang et al. (2022) found that the detection threshold for capsaicin (found in chili peppers and Sichuan pepper) did not significantly differ between older and younger adults. Similar results were observed in comparisons between young and old adults from the United States and Japan (Fukunaga et al. 2005; Wierenga et al. 2020). This discrepancy requires further investigation and verification, as there are limited studies on the effect of age on chemesthetic experience. Furthermore, some research has shown that younger adults can identify more test pieces with varied shapes than older adults, indicating that older adults have less tongue stereognosis capacity than younger people (Kawagishi et al. 2009).

### 2.2 Other Factors Influencing Sensory Abilities in Older Adults

Although impairment of sensory perception with age is common in the elderly population, studies have also shown that there is some individual variation in sensory loss in the elderly, which may be related to gender, occupation, environment, diseases, and psychological factors (Tseng et al. 2018). Most studies emphasized the challenge of identifying the ideal type and level of flavor because deficiencies associated with age may be quality dependent and differ in severity between people (Tan et al. 2018; Oleszkiewicz et al. 2020; Claus et al. 2022).

#### 2.2.1 Pathological Conditions and Unhealthy Lifestyle

Age‐related declines in sensory abilities can be exacerbated by various pathological conditions (Solemdal et al. 2012a, 2012b; Sulmont‐Rosse et al. 2015; Gao et al. 2020; Völter et al. 2021). Chronic diseases such as chronic sinonasal disease, rhinosinusitis, and nasal polyposis can impair olfactory function, leading to a reduced ability to detect and enjoy smells (Schubert et al. 2011). Additionally, cardiovascular disease and Alzheimer's disease have been linked to a decline in sensory perception, particularly affecting the ability to process complex sensory information (Völter et al. 2021). Poor oral health, including dental caries and periodontal diseases, can also affect taste perception, as these conditions can alter the oral environment and the sensitivity of taste receptors (Jonathan A 1999). Vision‐related conditions like glaucoma, diabetic retinopathy, age‐related macular degeneration, and cataracts are common among older adults and can lead to significant vision impairments (Whillans et al. 2016). HL can be caused by conditions such as otitis media, which can lead to degeneration and loss of auditory function (Davis et al. 2016). A study by McKee et al. (2018) found that HL in older adults is independently associated with various diseases, including arthritis, cardiovascular disease, diabetes, emphysema, high blood pressure, and stroke. Unhealthy lifestyle habits further contribute to sensory impairments. Smoking has been shown to increase the risk of HL, possibly due to its detrimental effects on blood circulation and the inner ear (Dawes et al. 2014). In contrast, moderate alcohol consumption has been found to be protective against age‐related HL, potentially due to its antioxidant properties (Dawes et al. 2014). Dietary factors, such as high carbohydrate and sugar intake, have also been linked to HL in older adults, possibly due to their impact on metabolic health and inflammation (Gopinath et al. 2010).

#### 2.2.2 Psychological Status

Many studies have highlighted the impact of psychological differences among older adults, particularly focusing on their quality of life, which is closely linked to social stimuli and activities (Fischer et al. 2009; Cavazzana et al. 2018; Olofsson et al. 2021). As Ellis et al. (2021) noted, poor quality of life, often characterized by social isolation, can lead to multiple psychological issues such as loneliness, depression, and anxiety, which in turn can result in sensory impairments. For instance, Robino et al. (2013) investigated 2956 older adults aged 60 and older and found that those with significant alexithymia traits had lower taste sensitivity than normal individuals. According to statistics, more than one in five people over the age of 60 years suffer from mental disorders such as depression, dementia, and anxiety (World Health Organization 2017), and these conditions can affect appetite and food intake.

The prevalence of depression varies globally and can manifest in different forms, including mild depression, bipolar disorder, and dysthymia. Social and biological factors, such as chronic illnesses and the long‐term use of certain medications, contribute to the development of depression in older adults (Rathod et al. 2022). Pause et al. (2001) found that individuals with depression have diminished olfactory ability, and a negative correlation exists between olfactory function and depression symptoms (Pause et al. 2001; Negoias et al. 2010). Alam et al. (2021) surveyed 400 older adults over 65 years in Bangladesh and found a strong association between depression and poor nutritional intake. Quandt et al. (2000) also noted that depression is often linked to loss of appetite and reduced food intake, particularly in women. Loneliness is more prevalent among older adults than in the general adult population (Ong et al. 2015). A study of 6786 community‐dwelling elderly Finnish people found a loneliness prevalence of 39.4% (Routasalo et al. 2006), and it was 56.63% among 3530 community‐dwelling adults aged 60 and older in the United States (Gerst‐Emerson and Jayawardhana 2015). In China, a study of 744 elderly participants residing in the community measured a loneliness prevalence of 26.2% (Zhong et al. 2019), while in Singapore, it was 51% (Chan et al. 2015). Ramic et al. (2011) investigated 200 older adults over the age of 65 years and found that those living alone consumed less food than those living with family, noting that loneliness was a significant predictor of anorexia nervosa in older adults. Whitelock and Ensaff (2018) conducted focus‐group discussions in the United Kingdom with 30 older adults aged 63–90 years and found that respondents reported a lack of motivation to cook due to having to eat alone. Tsofliou et al. (2020) surveyed 39 older adults and found that appetite was enhanced in the presence of a companion for meals.

Anxiety can lead to a decline in subjective well‐being and life satisfaction, particularly affecting cognitive functions related to food consumption (Taghiabadi et al. 2017; Malone and Wachholtz 2018; Divers et al. 2021). Paquet et al. (2003) observed 30 older adults aged 65–92 years and found that negative emotions such as anxiety, depression, and anger had a negative impact on food intake.

#### 2.2.3 Gender Differences

Gender differences in sensory abilities are evident among older adults, with men generally experiencing more sensory impairments than women. Several studies have shown that older men more frequently reported HL than older women, particularly among those aged 62–90 years (Pinto et al. 2014; Uchida et al. 2019; Reavis et al. 2022). For instance, Park et al. (2016) conducted a study involving 2818 men and 4132 women aged 50–85 years and found that men had significantly lower hearing thresholds at frequencies of 3 kHz, 4 kHz, and 6 kHz compared to women. This suggests that men are more susceptible to hearing impairments at these frequencies. Furthermore, a study by Correia et al. (2016) involving 3005 US adults aged 57–85 years revealed that men had significantly poorer sensory function in hearing, taste, and smell compared to women. These findings align with previous research, indicating a consistent pattern of gender differences in sensory abilities among older adults (Schneider et al. 2012; Pinto et al. 2014).

#### 2.2.4 Education, Income, and Occupation

Several studies have demonstrated that occupation and income can impact sensory abilities, particularly vision and hearing, in older adults (Andrade and López‐Ortega 2017; Tham et al. 2018; Wang et al. 2020). For instance, Andrade and López‐Ortega (2017) conducted a study involving 14,961 older adults aged 50–94 years in Mexico and found that women with lower levels of education were at a higher risk for hearing and visual impairments. Similarly, Tham et al. (2018) studied 3353 individuals aged 40 and older and observed that lower income and education levels were associated with bilateral vision impairment or blindness (*p* < 0.025).

Cao et al. (2021) used weighted multivariate logistic regression to analyze factors influencing sensory abilities among 15,176 Chinese seniors aged 65 and older. They found that vision impairment was prevalent and associated with age, marital status, economic status, educational level, occupation, and certain diseases. Adults with higher education and better self‐rated economic status had a lower risk of visual impairment, while those in traditional occupations (agriculture, forestry, animal husbandry, and fishing) and the unemployed had a higher likelihood of visual impairment, with all results being statistically significant (*p* < 0.05, *p* < 0.01). This suggests that lower education and economic status may lead to reduced health awareness and limited access to healthcare resources, thereby increasing the risk of sensory impairments.

Whillans et al. (2016) surveyed 2956 older adults over 60 years in the United Kingdom and found that visual stability varied with age, with those of higher social standing displaying better vision stability regardless of age.

In addition to occupation, the work environment and lifestyle also significantly affect sensory perception. Exposure to loud noises, such as those found in factories or from firearms, can lead to HL. Pinto et al. (2014) reported that older adults who worked in noisy environments were more likely to experience HL, and Yang et al. (2015) and Bowl and Dawson (2018) further supported this finding.

### 2.3 Impact of COVID‐19 on Sensory Impairment in Older Adults

COVID‐19 has significantly affected the global population, causing not only symptoms such as sore throat, headache, diarrhea, fatigue, muscle pain, shortness of breath, fever, and cough but also clinical issues like the loss of chemosensory function, vision, hearing, taste, and smell (Guo et al. 2020; Salcan et al. 2021; Nanjo et al. 2022; Chaaban et al. 2021). Studies have shown that many patients experience phantosmia (smelling odors without a source) and parosmia (distorted smell when a familiar odor is present) (Watson et al. 2010). Similar phenomena have been observed in taste (Chaaban et al. 2021). Additionally, COVID‐19 patients may develop sudden sensorineural hearing loss (SSNHL) and vision loss (Ricciardiello et al. 2021; Meng et al. 2022; van Rijssen et al. 2021; Swain and Pani 2021; Johansson et al. 2022).

While many patients regain their sense of taste and smell within weeks, approximately 10% report ongoing issues such as dysgeusia (distortion of basic tastes like bitter, sour, sweet, and salty), phantosmia, parosmia, hyposmia (reduced smell), anosmia (loss of smell), and decreased chemesthesis (reduced chemical sensations like the cooling of mint, the warmth of ginger, or the burn of chilies) (Burges Watson et al. 2021). These sensory dysfunctions can affect food preferences and reduce food intake and appetite, leading to weight loss (Fantozzi et al. 2020; Deer et al. 2022).

COVID‐19 impacts sensory loss through two possible mechanisms: receptor cell damage or infection and inflammatory cytokines (Iebba et al. 2021; Srinivasan et al. 2022). Older adults, who are more susceptible to inflammation, neurodegenerative diseases, and certain eye conditions, may face a higher risk of severe sensory loss compared to younger individuals (Hardan et al. 2021; Erausquin et al. 2021). Deer et al. (2022) found that men aged 60 and older consumed less total fruit and vegetables per day than recommended, highlighting the potential impact of COVID‐19 on the dietary quality of older adults. Furthermore, social distancing during lockdowns can exacerbate psychological issues and dietary patterns in older adults, further affecting their dietary quality (Piotrowicz et al. 2021). Visser et al. (2020) reported that 48.3% and 54.3% of older adults in the Netherlands aged 61 and above reduced their physical activity during the COVID‐19 pandemic, with older age groups at higher risk of undereating or weight loss.

In fact, recovery from COVID‐19 is also associated with “long COVID,” a term used to describe the persistence of various symptoms after acquiring a COVID‐19 infection, regardless of viral status (Raveendran et al. 2021). Numerous studies have indicated that long COVID is more prevalent among adult patients than among younger individuals (Sudre et al. 2021; Naidu et al. 2021; Jacobson et al. 2021). Long COVID not only affects the physical health of older adults but also has a negative impact on their mental health. Changes in lifestyle and prolonged sequelae can lead to more negative emotions, such as depression, loneliness, and anxiety (Shabbir et al. 2022). Nwachukwu et al. (2020) found that older adults in Canada (*n* = 762, aged 60 and above) scored lower on the Perceived Stress Scale (PSS), Generalized Anxiety Disorder 7‐item (GAD‐7) scale, and Patient Health Questionnaire‐9 (PHQ‐9) compared to younger individuals (≤ 25). Individuals aged 65 and older face a higher risk of death compared to younger age groups (Minnai et al. 2022). Although some studies have discussed strategies for older adults with long COVID, such as psychological support, consistent physiotherapy, and evaluation systems, there is a need for further investigation into age‐related sensory loss (Greenhalgh et al. 2021; Raveendran et al. 2021).

## 3 Evaluation Methodology

### 3.1 Sensory Evaluation Methods

**FIGURE 2:**
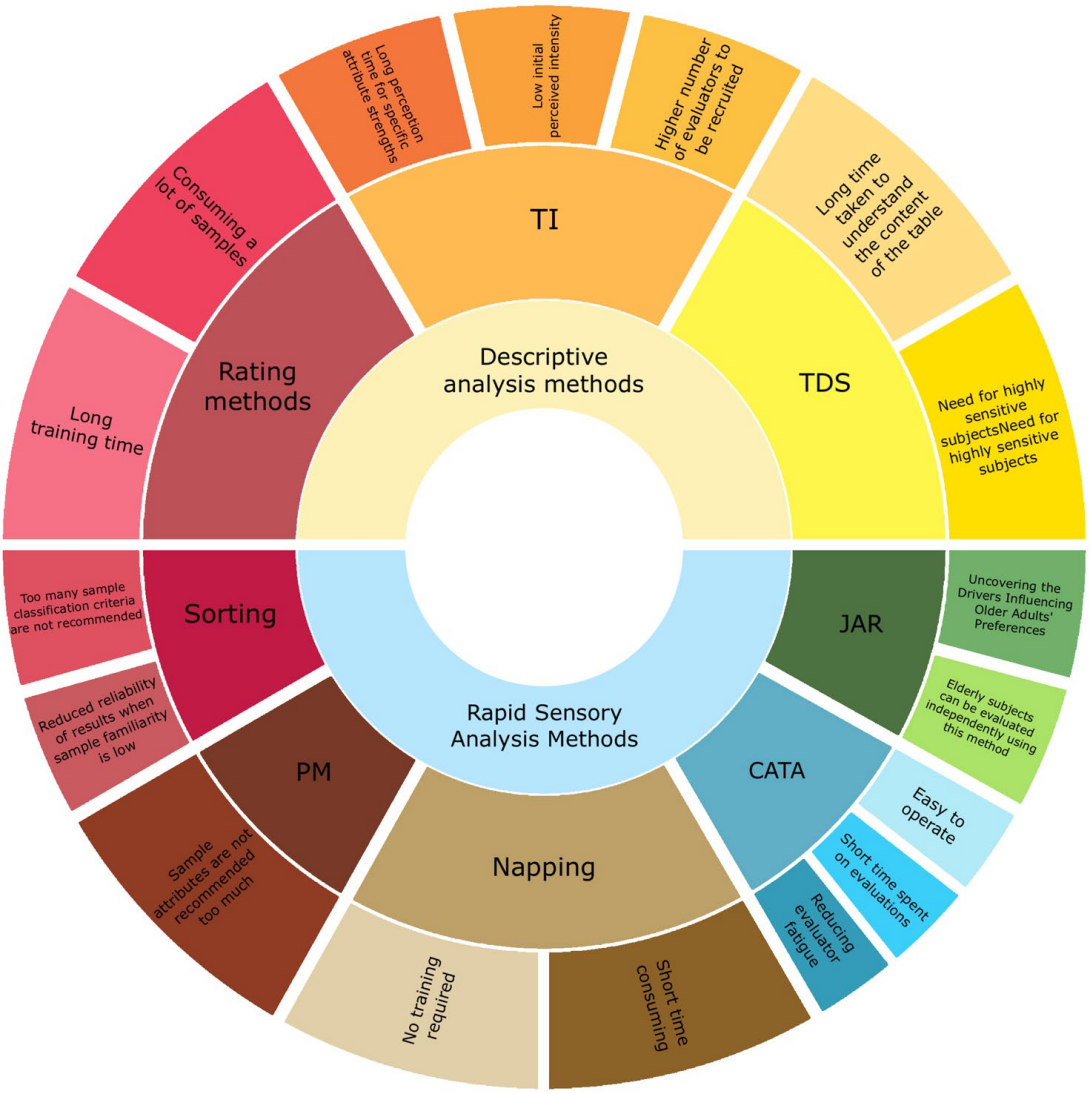
Feature of different sensory methods.

Aging may affect the sensory abilities of older adults, but the sensory appeal of foods has important implications for food selection as well as intake (Chaffee and Ross 2023). For the specific group of older adults, this section summarizes the sensory evaluation methods that have been commonly used in the last decade in terms of testing dimensions such as intensity, variance, and liking. Taking into account the specificities of older adults, we draw out the shortcomings and strengths of the application of these methods in older adults. In fact, there are many difficulties faced in sensory testing with older adults, which may be related to the sensory testing methods, such as some sensory evaluation methods being more oriented toward the younger population or may be related to the situation of older adults as a group itself (Cliceri et al. 2017; Scott et al. 2017). For example, the special characteristics of the elderly, short memory, physical fitness decline, ease of fatigue, and decreased sensitivity of smell and taste pose challenges. Based on these factors, we summarize the existing mainstream sensory evaluation methods and point out their strengths and weaknesses in the evaluation of the elderly, as shown in Figure 2.

#### 3.1.1 Descriptive Analysis Methods

Descriptive analytical methods are commonly used in sensory evaluation, where traditional descriptive tests are conducted by trained evaluation teams. More accurate food quality parameters such as appearance, color, flavor, taste, and texture of the food can be obtained by descriptive analytical tests (Methven et al. 2016). In the sensory evaluation of older adults, due to their special characteristics, the chosen research method and the design of the evaluation task should take into account the degradation of vision, smell, and taste, as well as the decline in comprehension. The questionnaire and experimental process should be designed in a targeted manner. A summary of the application of the descriptive method in sensory tests of older adults is presented in Table 2.

**TABLE 2:** Literature information on the descriptive method.

Method	Author	Publication date	Materials	Evaluators	Age	Article	Evaluation content
Intensity test	De J. N.	1996	Orange lemonade, strawberry jam, strawberry yogurt, grain, porridge, chocolate paste	Younger: 35 Older: 29	Younger: 22 ± 2 years old Older: 79 ± 6 years old	Effect of sucrose in breakfast items on pleasantness and food intake in the elderly	Sweet
9‐point intensity scale	S. Koskinen	2003	Oat bran product	Older: 50 Younger: 58	Older: 63–85 years old Younger: 18–34 years old	Flavor enhancement as a tool for increasing pleasantness and intake of a snack product among the elderly	Odor and taste intensity
100‐mm visual analog scale	Stefanie Kremer	2005	Chicken‐flavored white cream soup	Younger: 12 Older: 15 (9 normal sense of smell，6 impaired sense of smell)	Younger: 23 ± 3.4 years old Older: 68 ± 7.3 years old	Perception of texture and flavor in soups by elderly and young subjects. Journal of Texture Studies	Thickness, fluffiness, roughness, stickiness, swallow ability, creaminess, taste intensity, mushroom flavor, saltiness, chicken flavor
7‐point scale	S. Koskinen	2005	Hams	60 (54 females, 6 males)	61–86 years old	Intakes of cold cuts in the elderly are predicted by olfaction and mood, but not by flavor type or intensity of the products	Odor and taste intensity
100 mm horizontal visual analog scale	Stefanie Kremer	2007	Waffles	Younger: 16 Older: 22, 13 normal sense of smell, 9 impaired sense of smell	Younger: 18–35 individuals Older: 60–85 individuals	Differences in perception of sweet and savory waffles between elderly and young subjects	Fat, fluffiness, dryness, elasticity, swallow ability, aftertaste, sweetness, vanilla flavor, saltiness, cheese
15 cm line scale	Seo H S	2009	Green tea, coffee	Older: 30 normal sense of smell, 30 impaired sense of smell Younger: 30	Older: 60–75 years old (normal sense of smell), 62–79 years old (impaired sense of smell) younger: 18–30 years old	Effects of olfactory dysfunction on sensory evaluation and preparation of foods	Overall odor intensity
TI	Hutchings S. C.	2014a	Cashews	Younger: 16 Older: 16	Younger: 22–30, 26.3 ± 2.5 years old Older: 56–70, 63.2 ± 4.5 years old	Investigating changes in sensory stickiness perception with age	Differences in viscosity perception between elderly and young individuals
TI	Luckett C. R.	2016	Potato chips	68	Younger: 20–25 years old Middle‐aged: 40–45 years old Older: 65+ years old	Crispness level of potato chips affects temporal dynamics of flavor perception and mastication patterns in adults of different age group	Differences in crispness perception between elderly and young individuals
TI	Hitomi Sato	2022	NaCl solutions	Older: 31 (18 male, 13 female) Younger: 43 (21 male，22 female)	Older: 60–81 years old Younger: 21–39 years old	Differences in dynamic perception of salty taste intensity between young and older adults	Differences in salty taste perception between elderly and young individuals
TDS	A. Thomas	2018	Oral nutritional supplements (ONS)	62	60–75 years old	Temporal drivers of liking for ONS for older adults throughout the day with monitoring of hunger and thirst status	Attributes influencing likability
TDS	Hutchings S. C.	2014b	Nuts	Older: 20 Younger: 20	Older: 62.9 ± 4.8 years old Younger: 25.5 ± 2.5 years old	Temporal dominance of sensations: A comparison between younger and older subjects for the perception of food texture	Key attributes: hardness, crispness, stickiness, low oil

##### 3.1.1.1 Intensity Rating Methods (Rating Methods)

Quantification using rating methods typically requires the establishment of an evaluation team trained to achieve consistency in scoring and reproducibility of results (Aguiar et al. 2018). The main rating methods include categorical scales, linear scales, affective magnitude estimation scales, magnitude estimation scales, N‐point scales, and 100 mm horizontal visual analog scale (Methven et al. 2016). In sensory evaluation for older adults, these methods have been applied to products such as orange lemonade, strawberry jam, strawberry yogurt, cereal porridge, chocolate sauce (De Jong et al. 1996), oat bran products (Koskinen et al. 2003), cream soup (Kremer et al. 2005), ham (Koskinen and Tuorila 2005), waffles (Kremer et al. 2007b), green tea, and coffee (Seo and Hummel 2009).

Earlier studies have shown that older adults can use a variety of scales to perceive the overall odor intensity and overall flavor intensity of samples and can perceive and rate sweetness, elasticity, thickness, and flavor attributes such as creaminess and chicken flavor. In a study by De Jong et al. (1996), the authors used a 10‐point category scale to rate the sweetness of five types of products such as orange lemonade, strawberry jam, strawberry yogurt, cereal porridge, and chocolate sauce. They found that older adults (29 participants, mean age 79 ± 6 years) could distinguish well between the intensities of sweetness in different samples. Similarly, a larger group of individuals (50–60 participants, mean age 73.7 years) could accurately distinguish samples with differences in aroma intensity using either a 7‐point scale or a 9‐point scale (Koskinen et al. 2003; Koskinen et al. 2005). Notably, Seo and Hummel (2009), along with others, specifically recruited 30 young people with normal olfactory function, 30 elderly individuals, and 30 elderly people with olfactory dysfunction to assess the overall odor, flavor intensity, and bitterness of three different concentrations of green tea and coffee. Their study clearly demonstrated that individuals with olfactory dysfunction have a significantly reduced ability to perceive intensity (Seo and Hummel 2009).

Conducting intensity ratings using older adults is not limited to a single attribute; they can also evaluate multiple attributes simultaneously. Kremer et al. (2005) obtained the intensity of four attributes—sweetness, saltiness, cheesiness, and vanilla flavor—in a cream soup consumed by 15 older adults aged 60–85 years using a 100 mm horizontal visual analog scale. They also assessed six additional attributes: fatness, elasticity, dryness, fluffiness, swallowability, and aftertaste. In another study, Kremer et al. (2007b) evaluated the intensity of sweet/creamy, mushroom, chicken, savory, flavor intensity, thickness, and aftertaste in creamy soup waffles from 22 older adults aged 60–84 years using the same type of scale. The findings from Stefanie Kremer et al. indicated that the perception of creaminess, along with certain textures and flavors, was diminished in older adults.

The rating method demands a high level of competence from evaluators. Therefore, unless specifically required, potential subjects should undergo a survey and screening process to ensure they possess normal olfactory function, as assessed by the “Sniffin Sticks” test (Kremer et al. 2007b, 2005; Seo and Hummel 2009; Koskinen and Tuorila 2005), and normal cognitive abilities, as measured by the MMSE (Seo and Hummel 2009; Koskinen and Tuorila 2005). This process helps to identify any influences of subjects' backgrounds or illnesses on the study outcomes. Furthermore, the decline in perceptual abilities among older adults can complicate group training and research, potentially necessitating a larger sample size and a more extended training period to ensure data validity (Daute et al. 2021).

##### 3.1.1.2 Time‐Intensity (TI) Method

The TI method is a widely used dynamic descriptive analysis technique that focuses on scoring a single attribute of a sample. When multiple attributes are involved, results must be obtained through multiple tastings, which can significantly increase the cost of the experiment. Therefore, the TI method is not suitable for the evaluation of multiple attributes (Methven et al. 2016). In sensory evaluation involving elderly individuals, it has been applied to the evaluation of foods such as cashew nuts (Hutchings et al. 2014), crisps (Luckett et al. 2016), and salt solution (Sato et al. 2022).

In a study by Hutchings et al. (2014), the authors used the TI method to evaluate the dynamic viscosity perception of cashew nuts among16 older subjects (55–70 years old) and younger subjects (22–30 years old). It was found that the younger subjects reached the highest intensity earlier than the older subjects, confirming the decline in taste perception among the elderly. Another study using the TI method to test the temporal dynamics of crispiness perception involved 68 subjects from different age groups (young: 20–25 years, middle aged: 40–45 years, elderly: 65+ years) and showed that older adults perceived a significantly lower initial intensity of flavor compared to younger individuals (Luckett et al. 2016). A study on the savory flavor intensity in 31 elderly people (aged 60–81 years) revealed that while older adults could correctly identify salty flavors, their perception of specific intensities took longer. All of these studies highlight the effects of aging on taste perception (Sato et al. 2022).

When using the TI method for research, compensatory effects occur in the elderly population due to decreased chewing ability, so chewing time can be extended or the number of chews increased (Luckett et al. 2016). Additionally, because the TI method is specific to the evaluation of a single attribute, more subjects need to be recruited for the experiment to obtain more accurate group differences (Hutchings et al. 2014).

##### 3.1.1.3 Temporal Dominance of Sensations (TDS)

The TDS method is a dynamic descriptive analysis method that considers the dynamic processes of food digestion and flavor release (Oliver et al. 2018a) and allows for the simultaneous evaluation of multiple attributes in response to the dynamics of the food (Di Monaco et al. 2014). Usually completed by a trained panel, the TDS method can be completed by untrained subjects if the intensity of the attribute is not being evaluated (Di Monaco et al. 2014). In sensory evaluation of older adults, it has been applied to nuts (Hutchings et al. 2014), oral solutions (Thomas et al. 2018), and other products.

In a study by Hutchings et al. (2014), four types of nuts, almonds, roasted cashews, macadamia nuts, and roasted peanuts, were evaluated by 20 older and 20 younger people, and taste change curves were obtained for different age groups for different samples as the number of chews increased. The study proved that healthy subjects with a mean age of 62.9 ± 4.8 years were able to obtain clear TDS curves, as well as young people, and the attributes selected were consistent for both age groups. Thomas et al. (2018) evaluated different types of oral liquids with 62 older adults aged 60–75 years and found that the sensation of thirst in older adults was predominantly characterized by dryness attributes. Samples with higher protein content received higher preference due to their persistent nutty and coffee aromas, as well as their brief metallic taste and dryness.

These studies have validated the use of older adults as subjects to investigate the dynamics of sensory perception in the oral cavity, enabling them to identify which sensations are dominant (Albert et al. 2012). However, older subjects may take longer to understand the content of the table, which could be related to the decline in memory and visual abilities commonly experienced by older adults. Therefore, when designing experiments, it is important to consider suitable ways to present the table or other forms of information (Hutchings et al. 2014).

#### 3.1.2 Rapid Sensory Analysis Methods

With the development of sensory methods, rapid sensory analysis methods such as Sorting, Projective Mapping (PM), Just About Right (JAR), and Check All That Apply (CATA) have gradually emerged. These methods are completed by untrained evaluators, and the tests are simple and easy to understand, making them suitable for use in sensory evaluation of older adults. Combined with the preference tests, they can uncover the specific drivers of preferences that influence older adults. A summary of the application of these rapid methods in sensory tests for older adults is presented in Table 3.

**TABLE 3:** Literature information on rapid sensory methods.

Method	Author	Publication date	Materials	Evaluators	Age	Article	Evaluation content
Sorting	Scott N.	2017	Pudding	Younger: 60 Older: 60 (36 females, 24 males)	A: 18–30 years B: 60–88 years	Exploring the use of rapid profiling techniques for use in older adult populations	Food sensory characteristics
Sorting	Natalia Riquelme	2022	Dessert	87	> 60	Understanding older people perceptions about desserts using word association and sorting task methodologies	Views on desserts
Free sorting	Danny Cliceri	2017	Canned pea, canned sweet corn	Canned pea control group: 34 females, 21 males Canned sweet corn control group: 38 females, 20 males Experimental group: 166；148	Canned pea control group: 34 females, 21males; canned sweet corn control group: 38 females, 20 males; experimental group: 65–69 years old, 70–79 years old	Exploring salient dimensions in a free sorting task: A cross‐country study within the elderly population	Perception and preference
Projective Mapping	Scott N	2017	Pudding	Younger: 60 (42 females, 18 males) Older: 60 (36 females, 24 males)	A: 18–30 years old B: 60–88 years old	Exploring the use of rapid profiling techniques for use in older adult populations	Texture, flavor
QMA	Behannis Mena	2020	Snack	China: 21 (17 females, 4 males) Australia: 16 (13 females, 3 males)	China: 60–81 years old Australia: 65–79 years old	Exploring meal and snacking behavior of older adults in Australia and China	Food preferences
5‐JAR scale	Rutkowska J.	2022	Kefir	256 (158 females, 98 males)	65–76	Volatile composition and sensory profile of lactose‐free kefir, and its acceptability by elderly consumers	Perception and hedonic acceptability
5‐JAR scale	Ares G.	2010	Milk desserts	100	18–63	Comparison of attribute liking and JAR scales to evaluate the adequacy of sensory attributes of milk desserts	Overall liking, thickness, creaminess, sweetness, vanilla flavor rating
CATA	Scott N	2017	Pudding	Younger: 60 (42 females, 18 males) Older: 60 (36 females, 24 males)	A: 18–30 years B: 60–88 years	Exploring the use of rapid profiling techniques for use in older adult populations	Texture, flavor, acceptability
CATA	Regan E	2019	Oral nutritional supplements (ONS)	Experimental group: 80 Control group: 80	Experimental group: untrained，35 males, 45 females, > 65 years old Control group: untrained，35 females, 35 males, 18–35 years old	Exploring how age influences sensory perception, thirst and hunger during the consumption of oral nutritional supplements using the check‐all‐that‐apply methodology	The impact of thirst, hunger, and satiety on supplement preference
CATA/TCATA	Schumaker, M. R.	2019	Commercial Shiraz wine	105 (58 females, 37 males)	21–86 years	Influence of wine composition on consumer perception and acceptance of Brettanomyces metabolites using temporal check‐all‐that‐apply methodology	Aroma, taste, and mouth feel
CATA	Jaeger, S. R.	2020	Apple juice, red tea	636	19–68 years	Check‐all‐that‐apply four case studies with beverages	Similarities or differences in describing sensory product characteristics
CATA	Mahieu, B.	2021	French red wines, milk chocolates	A: 120, B: 147	A: 18–60 years B: 18–65 years	An investigation of the stability of free‐comment and check‐all‐that‐apply in two consumer studies on red wines and milk chocolates	The stability of the same product assessed using Free‐Choice Profiling (FC) method versus Check‐All‐That‐Apply (CATA) method
CATA	Regan E	2021	ONS	80	51 (65–74 years old)， 29 (> 75 years old)， 74 ± 8 years old (65–97) 35 males, 45 females	Exploring how age, medication usage, and dentures effect the sensory perception and liking of oral nutritional supplements in older adults	Hunger, satiety, food cravings, and thirst

##### 3.1.2.1 Sorting

The sorting method, with its roots in psychology, is a relatively new approach in sensory evaluation (Dominique et al. 2018). This method requires subjects to group samples based on their overall similarity and typically involves 9–15 trained subjects or 9–98 untrained subjects (Fleming et al. 2015). It has been applied to the sensory evaluation of foods such as peas and corn (Cliceri et al. 2017), puddings (Scott et al. 2017), and desserts (Riquelme et al. 2021) in studies involving older adults.

In a study by Scott et al. (2017), seven puddings were evaluated using the sorting method. The results demonstrated that untrained older subjects (aged 60–88 years) could effectively sort the samples according to the degree of similarity, with 75% of the older adults completing the sorting experiment correctly. In the evaluation of the desserts, all older adults expressed a clear opinion on all 12 desserts (Riquelme et al. 2021). In the study of canned peas and sweet corn, 10 types of canned peas and 8 types of canned sweet corn were evaluated, respectively, using the categorization method, and older adults (over 65 years) still demonstrated good classification skills (Cliceri et al. 2017).

However, the sorting method can be influenced by the familiarity of the samples. Cliceri et al. (2017) found that for samples with a high level of familiarity, the categorization result plots were highly reliable across all age groups. In contrast, in the evaluation of samples with low familiarity, there were differences in the evaluation criteria used by the subjects, which led to a decrease in the reliability of the results. Therefore, it is recommended to conduct experiments based on the familiarity of the test population (e.g., gender, age) with the samples (Cliceri et al. 2017). Additionally, some elderly subjects were unable to categorize the samples based on multiple criteria. For this reason, the authors suggest that selecting a group of samples with relatively small differences for further testing (Scott et al. 2017).

##### 3.1.2.2 Projective Mapping

PM has a long history in the field of psychology, and it is a relatively new evaluation method in the sensory field. The PM method is completed by at least 20 subjects, who are generally untrained consumers (Mena et al. 2020). Subjects place the samples on corresponding coordinate paper based on the overall similarity of the samples (Dominique et al. 2018). Qualitative multivariate analysis (QMA) is a derivative of the PM method, and its basic process and requirements are similar to PM (Daute et al. 2021; Mena et al. 2020; Dehlholm et al. 2012).

Scott et al. (2017) recruited 60 older subjects (aged 60–88 years) and 60 younger subjects (aged 18–30 years) to evaluate the similarity of seven puddings and two replicate samples using the PM method. The results showed that older subjects could correctly place the duplicate samples on the coordinate paper. Comparison of the result plots showed that there was a correlation of 0.89 between the evaluation results of the older subjects and the results of the younger subjects (Scott et al. 2017). Mena et al. (2020) recruited 21 Chinese (aged 60–81 years) and 16 Australian (aged 65–79 years) older subjects to evaluate jerky, chocolate, berries using the QMA method. The criteria for placement were *x*‐axis: “every day” to “indulgence,” *y*‐axis: “likely to eat” to *y*‐axis: “likely to eat” to “unlikely to eat.” The results clearly concluded that Chinese older adults have healthier eating habits than Australian older adults. It was also found that foods that met the criteria of “ready‐to‐eat” and moderate size were preferred more by the older population.

**TABLE 4:** Summarized literature on food compensation strategies.

Modal	Elderly population			Decline/impairment of sensory perception				Food system	Compensatory strategies	Effect of compensatory strategies
	Sample size	Age	Living condition	Taste	Order	Auditory	Version			
Taste										
Sweet	*N* = 191	70–75	—	—	—	—	—	A variety of foods	Sugar	Positive
	*N* = 35	59–88	—	—	—	—	—	Apple juice	Sweetness	Positive
	*N* = 36	82.8 (F)78.8 (M)	Nursing home	Yes	Yes	—	—	Peach juice	Sweetness	Positive
	*N* = 25	79 ± 6	Nursing home	—	—	—	—	Orange lemonade, strawberry jam, strawberry yogurt	Sweetness	Positive
Sour	*N* = 52	60–85	Community dwelling	—	—	—	—	Desserts	Sour	Positive
	N = 76	64–97	Clinical Rehabilitation Institute	Yes	Yes	Yes	—	Functional drink	Lower sourness	No effect
Salt	N = 35	62–83	Nursing home	—	—	—	—	Meat	Enhance salt	Positive
	*N* = 84	65.2–68.5	Community dwelling	Yes	Yes and No	—	—	Mashed potatoes	Salt	No effect
	*N* = 114	63–102	Nursing home	Yes and no	—	—	—	Finger foods	Salt	Positive
	N = 52	71.1 ± 4.6	Community dwelling	Yes	—	—	—	Lunch meal and evening meal	Sauce	Positive
	*N* = 97	> 65	Nursing home	—	—	—	—	Cooked meal	Flavor	No effect
Smell	*N* = 71	65–93	Nursing home	—	—	—	—	Oat bran frankfurters	Spices	Positive
	N = 71	> 65	Nursing home	—	—	—	—	Cooked meals	Flavor powders	Positive
	*N* = 104	79–101	Nursing home	—	—	—	—	Appetizer	Flavor	Positive
	N = 84	65.2–68.5	Community dwelling	Yes	Yes and no	—	—	Gravy and both stews	Flavor	No effect
Version	N = 71	65–93	Nursing home	—	—	—	—	In‐between meals	Flavor	Positive
Auditor**y**	*N* = 30	66–75	—	—	—	—	—	Nursing care foods	Auditory	Positive
Taste–smell	*N* = 100	65–93	Community dwelling	—	—	—	—	Lemon‐flavored beverage base	Sucrose and citric acid	Positive
	N = 52	60–85	Living independently	Yes	—	—	—	Desserts	Cherry flavor and cream flavor	Positive
	N = 25	65–83	Community dwelling	—	—	—	—	Meal	Tastes and flavors	Positive
Taste‐texture	*N* = 29	84.4 ± 4	Nursing home	—	—	—	Yes and no	Main meals	Tastes and flavors	Positive
	*N* = 62	70 ± 5	—	Yes	Yes	—	—	Yogurt	Sweetness and hardness	No effect
	*N* = 37	60–81	Community dwelling	Yes	Yes	—	—	Meal and snacking	Flavor–texture	Positive
Taste–appearance	*N* = 122	79.4 ± 1.1	—	—	—	—	—	Maize soup	Vision and taste	Positive
	N = 37	60–75	—	—	—	—	—	Bread	Color and taste	Positive
Appearance–texture	N = 37	65–101	Nursing home	—	—	—	—	A variety of foods	Color and hardness	Positive
	*N* = 20	51–64& > 80	Nursing home	—	—	—	—	Ice cream and desserts	Color and hardness	Positive

Some of the elderly subjects were unable to place the samples using the PM method considering multiple attributes at the same time. It is suggested that the subjects focus on one attribute at a time and be guided through the experiment in multiple sessions (Scott et al. 2017). As there are also differences within the elderly, such as age, health status, and nutritional need, it is recommended to consider individual differences in elderly subjects when conducting research (Mena et al. 2020).

##### 3.1.2.3 Napping

Napping, developed by Pagès (2003), is a specific variant of the PM method. It is a relatively recent and rapid sensory description technique used for sensory evaluation. Napping involves projecting food samples onto a two‐dimensional plane (typically sized 60 × 40 cm), where products with similar attributes are placed closer together and those that are more dissimilar are placed further apart (Reinbach et al. 2014; Pickup et al. 2017). This method has been applied in the context of the UFP (ultra‐flash profiling) for the sensory evaluation of confectionery products (Reinbach et al. 2014; Pickup et al. 2017). Napping is often used in conjunction with UFP and requires data analysis using multiple factor analysis (MFA). It has been utilized in experiments involving confectionery gels (Frantz Lairy Obas et al. 2021), caramel corn (Mayhew et al. 2016), and green tea (Kim et al. 2013), among others. Typically, napping is suitable for experiments conducted by 10–12 untrained evaluators. However, Liu et al. (2016) found that method‐trained groups could group wines more clearly in their study of small sensory differences, suggesting that training in methods and samples may lead to more reliable results.

In addition, the variation and accuracy of the results may be influenced by the familiarity of the samples. In an experiment by Park et al. (2014), a group of people aged 34–68 years (mean age 51.6 years) participated in napping sensory evaluations of traditional Korean biscuits. They found that younger consumers tended to place biscuits with strong odors and ginger flavors together, both of which may be perceived as pungent. Older consumers, on the other hand, placed two types of biscuits from the same company, produced according to traditional methods, closer together, possibly due to their greater exposure to these traditional biscuits. There were also differences in the frequency of use of perceptual and sensory terms between older and younger consumers, with terms like “very oily” used more often by the older group and “hard” by the younger group. This experiment suggests that napping can reveal sensory differences among consumers. Napping is less time consuming than traditional analytical methods, making it suitable for the elderly population who are prone to fatigue. It can also better highlight differences between samples (Oliver et al. 2018b; Liu et al. 2016).

##### 3.1.2.4 Just About Right

JAR scale is one of the commonly used methods in consumer sensory evaluation to obtain the consumers' acceptance of an aspect or overall perception of a sample (Ares et al. 2009). The JAR method is often used in conjunction with preference testing and is generally completed by a minimum of 25 untrained evaluators (Lee et al. 2021). In sensory evaluations involving older adults, it has been applied in studies of kefir (Rutkowska et al. 2022), milk desserts (Ares et al. 2009), and cheese (Esmerino et al. 2015).

Rutkowska et al. (2022) investigated the perception and acceptance of lactose‐free kefir (LFK) products among older consumers (aged 65–76 years) using a JAR scale. A 5‐point JAR scale (1 = *too little*, 2 = *slightly too little*, 3 = *just right*, 4 = *slightly too much*, 5 = *too much*) was used in the study to rate the sweetness, tartness, and refreshing effect of kefir. The study showed that 63% of the elderly subjects rated the sweetness of LFK as “just right” and 73% rated the acidity of LFK as lower than that of traditional kefir. Nearly 50% of the subjects rated LFK as “like it a lot,” indicating that both acidity and sweetness are attributes that influence consumer preference. Ares et al. (2009) used a 5‐point JAR scale to evaluate the overall preference, creaminess, thickness, sweetness, and vanilla flavor of eight milk desserts among subjects aged 18–63 years. The results showed that subjects could accurately assess the relationship between each attribute of the samples and their level of preference using the JAR scale, and the samples that were closest to the ideal were identified.

With the development of sensory methods, the JAR method is considered a good judgment method because subjects can independently use this method to rate different attributes. The JAR method can help subjects think about the reasons for high or low preferences for samples, suggest deficiencies, and even provide suggestions (Ares et al. 2009). This can be very helpful in uncovering the factors that influence the preference drivers of older adults.

##### 3.1.2.5 CATA Method

The CATA method is a relatively recent approach for rapid sensory analysis. It is typically conducted by untrained subjects. A study by Ares et al. (2014) clarified that the number of subjects should be limited to 60–80 if there are significant differences between the evaluation samples in order to obtain reliable results. During the experiment, subjects are required to tick all the words that can characterize the samples from the list of descriptors provided to them (Jaeger et al. 2020). This allows researchers to identify the ideal product and product characteristics in the minds of the subjects. In the elderly population, this method has been applied in the sensory evaluation of food products such as pudding (Scott et al. 2017), wine (Schumaker et al. 2019; Mahieu et al. 2021), apple juice, and iced tea (Jaeger et al. 2020) and oral nutritional supplements (ONS) (Regan et al. 2019, 2021).

Older adults can identify sample differences and obtain product characteristics through the CATA method, which offers advantages in sensory evaluation for this age group. Scott et al. (2017) evaluated seven types of puddings by 60 older adults (aged 60–88 years) and 60 younger adults, successfully obtaining the corresponding product characteristics with RV coefficients of up to 0.98 for both age groups. This suggests that the CATA method is applicable for sensory evaluation in older adults and yields convincing results. Regan et al. (2019) characterized the sensory attributes of different flavors of ONS using 80 older adults and 80 young adults. They obtained the characteristics of the samples while enhancing the understanding of the preference factors of older adults. In Schumaker et al. (2019)'s sensory evaluation of wine, the CATA method was used to compare the aroma of seven wine samples, and elderly subjects (over 60 years old) clearly distinguished the differences in aroma among all the samples. Similarly, Jaeger et al. (2020) confirmed the ability of older adults (over 60 years old) to accurately complete the evaluation task for apple juice and iced tea with varying levels of acidity and sweetness.

Older subjects differed from younger subjects in the frequency of selection of an attribute. In a study of oral supplements of different protein types, younger subjects more frequently chose “hazelnut,” “metal,” “milk,” and “vanilla” to describe the nonconcentrated protein type of supplements. For protein concentrate nutrients, older subjects (mean age 73.7 ± 7.9 years) frequently chose “chocolate” to characterize them (Regan et al. 2019). Additionally, older subjects (29 participants, aged over 75 years) still frequently chose attributes to characterize the samples when different age groups of older adults were the subjects (Regan et al. 2021). This study also demonstrates the applicability of the CATA method in sensory evaluation of elderly subjects of different ages.

Overall, the CATA method is simple to operate, the evaluation consumes relatively little time, and it can effectively avoid issues such as reduced attention and increased fatigue, making it an ideal method for obtaining older adults' perceptions of sample features.

##### 3.1.2.6 Methods for Assessing Appetite and Intake in Older Adults

Appetite and food intake are crucial for maintaining the nutritional balance of older adults (Regan et al. 2019), and changes in these factors are important indicators of their physical health (Methven et al. 2016). When studying these issues, it is essential to consider the decline in smell and taste that often occurs with aging. Therefore, methods that are less fatiguing, quick, and simple should be used (Allen et al. 2014). Current methods applied to appetite and food intake in older adults include the 5‐point visual scale (Koskinen et al. 2003), the 7‐point hedonic scale (Koskinen et al. 2005), 100 mm visual analog scale (100 mm VAS) (Regan et al. 2019, 2021), [0–10] continuous scale (Thomas et al. 2018), and TDS (Thomas et al. 2018).

In an earlier study, De De Jong et al. (1996) recruited 33 young adults with a mean age of 23 ± 2 years and 25 older adults with a mean age of 82 ± 5 years. They used a crossover design to analyze the intake of five products, including orange lemonade, strawberry jam, strawberry yogurt, cereal porridge, and chocolate sauce, with varying sucrose concentrations. The study found that different sucrose concentrations did not significantly affect intake in either older or younger subjects (De Jong et al. 1996).

In a study by Koskinen et al. (2003) on the impact of flavor enhancement on intake, a group of 50 older subjects (aged 63–85 years) and 58 younger subjects (aged 18–34 years) were tested using a 5‐point visual scale. They evaluated the intake of three cups of regular aroma concentration oat bran and three cups of intensely flavored oat bran. Each score from 1 to 5 represented 0% (tasting sample only), 25%, 50%, 75%, and 100% consumption, respectively. The results showed that the mean intake of older subjects was numerically lower than that of younger subjects, but there was no significant difference (Koskinen et al. 2003).

A cross‐sectional study combined with a 7‐point hedonic scale was used to divide 60 older subjects (aged 61–86 years) into two groups to evaluate four types of commercial ham and three types of homemade ham with enhanced flavors (Koskinen et al. 2005). The study asked for a first preference rating of all samples received, followed by a rating of the samples consumed to obtain differences in intake. The authors found that a decrease in olfactory ability (as represented by poor health, age, and emotional state) might actually increase intake, especially in commercial hams (Koskinen et al. 2005).

In recent years, Regan et al. (2019, 2021) recruited 80 elderly subjects to use 100 mm VAS to assess the effects of ONS on appetite. The elderly subjects in the study were > 65 years of age. The study effectively demonstrated that ONS can lead to decreased appetite in older adults, potentially influenced by various physiological, psychological, and digestive factors (Regan et al. 2019, 2021).

The study by Thomas et al. (2018) recruited 62 older participants (aged 60–75 years), and used a [0–10 scale combined with the TDS method. They analyzed the factors affecting food intake using the mean values of hunger, desire to eat, desire to consume, and 100% satiety as references. The study found that “the eating environment (e.g., laboratory vs. home) affects intake,”“continuous drinking has less effect on hunger and more on thirst,” and “appetite is greater in the afternoon than in the morning” (Thomas et al. 2018).

Overall, first, when designing studies related to appetite and intake, it is often necessary to consider a balanced crossover sequence to eliminate experimental error. Second, due to the influence of subjects' own health conditions, a larger number of subjects may need to be involved to obtain significant and accurate results (Methven et al. 2016).

## 4 Measures and Effects of Food Sensory Compensation in Older Adults

**FIGURE 3:**
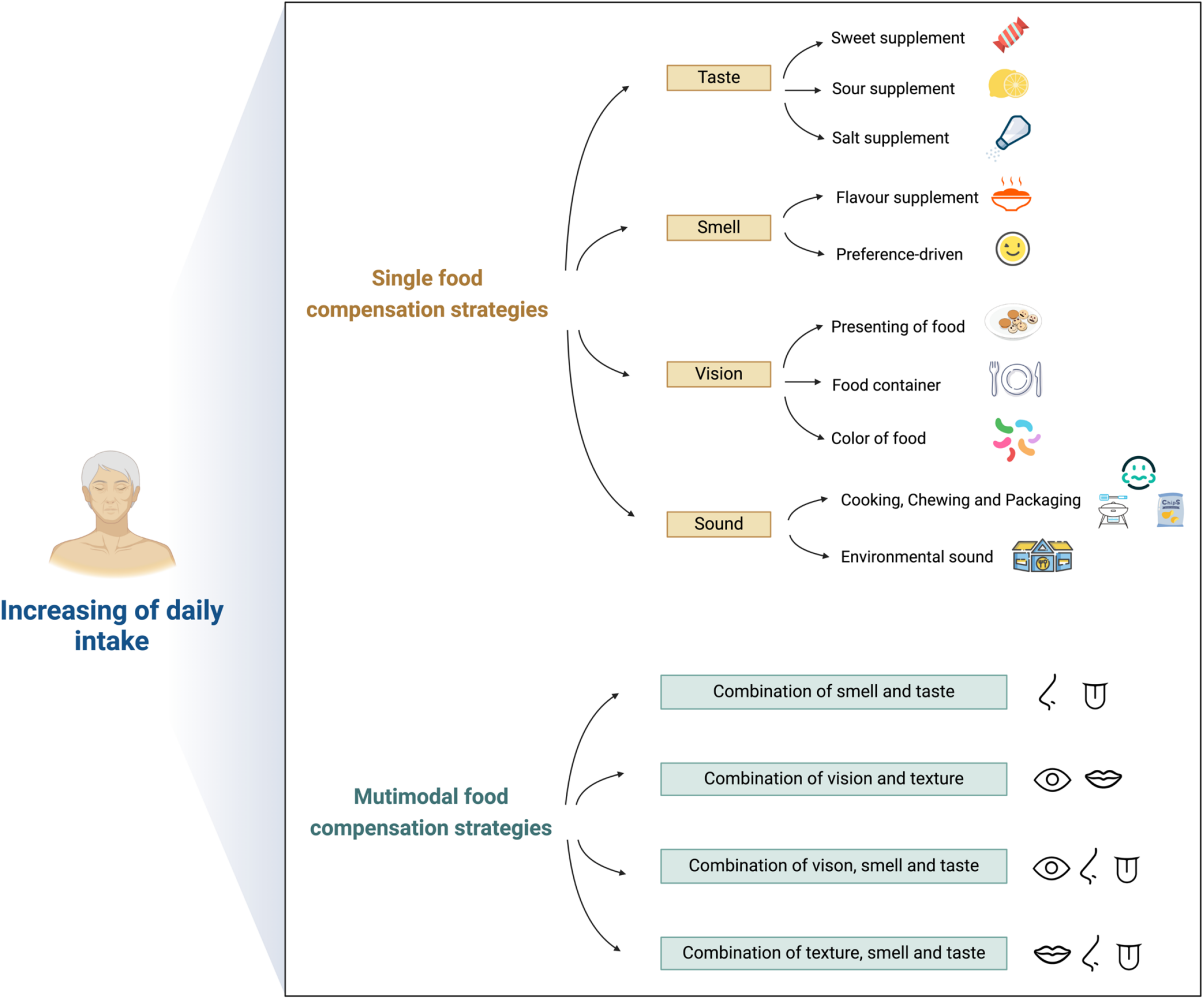
Sensory improvement strategies on older adults' daily intake.

The deterioration of sensory perception is a pivotal factor influencing food preferences and intake in older adults. As discussed in Section 2, this decline can lead to reduced dietary intake, contributing to issues like weight loss and frailty. To counteract these effects, it is essential to explore and implement food sensory compensation strategies that enhance older adults' enjoyment and consumption of food (Figure 3). This section provides an in‐depth analysis of various compensation strategies targeting different sensory perceptions, highlighting their potential to improve older adults' food preferences and intake (See details in Table 4).

### 4.1 Compensatory Strategies for Single Sensory

Overall, compensation strategies targeting taste and aroma show potential in increasing older adults' preference and intake of food. By increasing the intensity of one or several sensory attributes in food or by enhancing the dining environment, the acceptability of food for older adults can be effectively improved (Song et al. 2016). However, the effectiveness of these strategies may vary depending on individual preferences and the specific types of food involved, necessitating personalized adjustments in practical applications to better meet the dietary needs of older adults (Thomas et al. 2021). In addition to increasing sugar concentration, using sweeteners such as sugar alcohols and artificial sweeteners can enhance sweet taste perception without adding calories. These alternatives can satisfy older adults' desire for sweetness without increasing the risk of diabetes.

#### 4.1.1 Sweet Taste

It has been found that sweet taste compensation strategies indicate that as age increases, older adults' taste thresholds for sweetness also rise (Schiffman et al. 1981; Easterby‐Smith et al. 1994; Methven et al. 2012), so increasing the concentration of sweetness in food can enhance their preference and intake to a certain extent (Schiffman et al. 1981; Toffanello et al. 2010; Pouyet et al. 2015). Studies have found that older adults tend to prefer beverages with higher sugar content, such as apple juice and peach juice (Kozlowska et al. 2003; Laureati et al. 2008). However, some studies have pointed out that simply increasing the concentration of sweetness does not always significantly increase food intake, which may be related to older adults' tolerance to sweetness, other sensory attributes of the food, and their overall dietary habits (De Jong et al. 1996). Therefore, when applying sweet taste compensation strategies, it is necessary to consider other factors comprehensively to achieve the best results.

#### 4.1.2 Sour Taste

The results of research on sour taste compensation strategies are relatively complex. On one hand, some studies have shown that adding low concentrations of sour taste components (such as cherry flavor) to desserts can increase older adults' preference and intake of food (Kremer et al. 2007a), which may be due to the synergistic effect of sour taste with other flavors, making the overall taste of the food richer and more pleasant. On the other hand, other studies have found that enhancing sour taste in beverages does not always effectively promote intake in older adults (Arganini et al. 2013), which may be related to their sensitivity to sour taste, preferences, and other sensory attributes of the beverages. In addition, there are differences in the acceptance of sour taste among different individuals, so when applying sour taste compensation strategies in practice, it is necessary to fully consider the individual differences of older adults and the specific types of food to achieve the best compensation effect.

#### 4.1.3 Salty Taste

Research on salty taste compensation strategies indicates that older adults have a higher taste threshold for saltiness, and appropriately increasing the saltiness in food can improve their preference for food to a certain extent (Pangborn and Braddock 1989; Dermiki et al. 2013). For example, studies have found that increasing the saltiness in mashed potatoes and meatloaf can enhance older adults' preference for these foods (Kremer et al. 2014). Additionally, studies have found that adding sauces to food (Pouyet et al. 2014; Kimura et al. 2019; Appleton 2018; Best and Appleton 2011) leads to older subjects always preferring food with added sauces over standard samples, with the enhancement of saltiness effectively increasing their preference and intake. However, some studies have pointed out that adding seasonings or monosodium glutamate (MSG) when cooking meat does not always significantly increase older adults' dietary intake (Essed et al. 2007), which may be related to their sensitivity to saltiness, other sensory attributes of the food, and their overall dietary habits. Moreover, excessive intake of salt can have adverse effects on the health of older adults, so when applying salty taste compensation strategies, it is necessary to find a balance between improving food preference and ensuring health, while also considering the individual differences of older adults and the specific types of food.

#### 4.1.4 Aroma

Aroma compensation strategies play an important role in increasing older adults' preference and intake of food. Research has shown that adding spices and seasonings to food, especially natural spices such as herbs, onions, and garlic, can significantly enhance older adults' interest and intake of food (Thomas et al. 2021; Kimura et al. 2019; Best and Appleton 2011; van den Heuvel et al. 2020). For example, studies by van den van den Heuvel et al. (2018) and Henry et al. (2003) have found that adding spices can increase the attractiveness of food, making older adults more willing to consume it; adding cheese flavor to sausages is also favored by older adults (Elsner et al. 1998). Additionally, enhancing the aroma of food can also increase older adults' hunger and daily dietary intake (Mathey et al. 2001). In the study by Pouyet et al. (2015), soft bread and aubergine sauce samples were used as blank control samples, and flavorings such as lemon, roasted garlic, salt, and pepper were added, showing that older adults had a higher intake of the flavor‐enhanced samples. However, Koskinen et al. (2003) found that adding redcurrant aroma to oatmeal yogurt and enhancing the samples' aroma showed a positive correlation with the preferences of older subjects, but aroma enhancement did not directly increase intake in older adults. There are also studies pointing out that enhancing aroma in stews does not always significantly improve older adults' preference and intake of food (Kremer et al. 2014), which may be related to other sensory attributes of the food and the individual differences of older adults. Therefore, when applying aroma compensation strategies, it is necessary to consider the multiple sensory attributes of food and the specific needs of older adults comprehensively to achieve the best effect.

#### 4.1.5 Vision

Visual compensation strategies play a vital role in the diet of older adults. With the decline of olfactory and gustatory functions, the importance of vision in food perception is increasingly highlighted (Wijk et al. 1999). Research has shown that older adults have better discrimination and higher preferences for bright colors such as red and yellow, so enhancing the visual impact of food by adding food coloring can effectively promote their food intake (Schiffman 2000; Garber et al. 2001). For example, colorful foods such as ice cream and cheesecake are more favored by older adults (Wendin et al. 2021). In addition, using larger tableware and increasing the portion size of food can also significantly increase food intake (Wansink and van Ittersum 2003; Wansink et al. 2005). However, there are relatively few studies on the impact of single visual compensation on the diet of older adults at present, and more related research is urgently needed in the future to further explore the mechanism of action and application strategies of visual factors in the diet of older adults, providing a scientific basis for improving their dietary experience and nutritional intake.

#### 4.1.6 Hearing

Auditory compensation strategies play an undeniable role in the perception of food flavor. Research has shown that various sounds related to food, such as the sound of unwrapping packaging, the sound of food preparation, and the sound of chewing, can affect older adults' perception of food taste (Wang et al. 2019; Endo et al. 2017). For example, providing older adults with prerecorded chewing sounds can improve their perception of the taste of unevenly textured food, while playing fake chewing sounds that do not match the texture of the food may change their perceptual experience. In addition, sound stimuli in the environment, such as music, can also cause emotional changes, which in turn affect food preferences and dietary intake (Guéguen et al. 2008; Crisinel et al. 2012). When music with a sweet atmosphere is played, it can enhance people's perception of the sweetness of food, while music with a sad atmosphere may increase the perception of bitterness (Guedes et al. 2023). This indicates that the emotional response elicited by sound can regulate people's sensory evaluation of food. Although there are relatively few studies on auditory compensation strategies at present, their potential in improving the dietary experience and promoting healthy eating in older adults is worth further exploration.

### 4.2 Implementation and Effectiveness of Multimodal Food Compensation Strategies

Multimodal food compensation strategies have shown significant advantages in increasing older adults' preference and intake of food. The effectiveness of single‐sensory compensation strategies is limited, while multimodal strategies can compensate for the insufficiency of single‐sensory perception through the interaction and synergistic effects between different senses, thereby more effectively enhancing the dietary experience of older adults (Wang et al. 2022). In recent years, some studies have explored food compensation strategies for older adults from multiple dimensions such as food odor, appearance, and texture simultaneously (Klosse et al. 2004; van der Meij et al. 2015). Improving the taste, texture, and appearance of food through multimodal strategies can enhance consumers' sensory pleasure, increase their preference for food, and thus promote food intake. The following sections will focus on reviewing the impact of four types of sensory interactions, namely, taste–aroma, taste and aroma–texture, appearance–taste, and appearance–texture, on older adults' food preferences and intake.

#### 4.2.1 Taste and Aroma

The olfactory and gustatory systems are interconnected and activated in the same areas of the brain, so adding aroma and taste modifiers to food can enhance older adults' preference and intake of food (Bellisle et al. 1991; Henry et al. 2003; Mathey et al. 2001; Gagnon et al. 2014; Biswas and Szocs 2019). For example, sweetness and specific flavor compounds increase older adults' preference for and intake of ONS (Lester et al. 2022). Adding sucrose and citric acid to beverages at appropriate concentrations can significantly enhance older adults' liking for the beverages (Murphy and Withee 1986); adding two different aroma compounds, cream and a subtle hint of cherry, to desserts can also significantly increase the preference of most older adults (Kremer et al. 2007a). Meals fortified with a variety of flavors and odors (herbs, spices, onions, and garlic) enhance food liking, thereby increasing food intake (Thomas et al. 2021). Appleton (2009) and Best and Appleton (2011), respectively, investigated whether adding sauces to food could increase older adults' food intake and showed that consuming meals fortified with sauces containing a variety of flavors and aromas resulted in a significant increase in older adults' energy, protein, and fat intake. However, the degree of preference for different flavors among older adults may vary depending on their olfactory and gustatory abilities, and they may only show a higher preference for certain specific flavors in specific food systems (Kremer et al. 2014), which suggests that when applying taste–aroma compensation strategies, it is necessary to fully consider the individual differences of older adults and the specific types of food to achieve the best compensation effect.

#### 4.2.2 Taste–Aroma and Texture

Texture and flavor are key factors affecting older adults' food choices, and enhancing the texture and nourishing flavor of food simultaneously can effectively improve their preference for food, thereby promoting their food intake (Mena et al. 2020; Forsberg et al. 2022). For example, finger snacks developed for Swedish older adults with motor difficulties were made softer by adding whey protein, soy protein, and fat, and off‐flavors in the sauce were reduced with sweet, sour, and bitter flavors, providing an excellent taste experience for these older adults (Forsberg et al. 2022). However, whether changes in the sensory characteristics of food can effectively enhance older adults' preferences also depends on the characteristics of the food itself and the individual differences of older adults. For example, in yogurt, texture has less impact on older adults (Kalviainen et al. 2002), but in cream soups and waffles, improvements in texture and flavor are extremely important for older consumers (Kremer et al. 2005, 2007b). Recent studies have also found that adding plum sauce (as a lubricant, while enhancing sweet and sour flavors) has no significant effect on older adults (Behannis et al. 2022). Therefore, when applying such compensation strategies, it is necessary to consider the multiple sensory attributes of food and the specific needs of older adults comprehensively to achieve the best compensation effect.

#### 4.2.3 Appearance and Taste–Aroma

The color, shape, and other appearance characteristics of food have an important impact on consumers' attraction, and their combination with taste and aroma can further enhance the attractiveness of food (Piqueras‐Fiszman and Spence 2014). Research has found that pink and red are most strongly associated with sweet taste, yellow and green with sour taste, white and blue with salty taste, and brown/black and purple with bitter taste (Spence et al. 2015). For example, using ingredients such as cilantro, red peppers, bacon, and sour cream to improve the appearance and taste of high‐energy corn soup can significantly increase older adults' preference for it, thereby promoting their intake (Zhou et al. 2021). In addition, improving the appearance (such as darker color) and taste (with the typical smell and flavor of bread) of bread can also enhance older consumers' satisfaction and preference for bread (Moretton et al. 2023). This indicates that when people choose food, they not only pay attention to its taste and smell but also place great importance on its appearance characteristics. For example, strawberry and chocolate beverages are favored for their sweet taste and bright colors (Zellner et al. 2014), and cakes are favored for their unique shape. The visual stimulation of color may trigger a preference for expected taste perceptions.

#### 4.2.4 Appearance and Texture

The appearance and texture of food not only attract consumers' attention but also affect their preferences and intake. For example, biscuits with smooth surfaces and crispy textures are more favored by consumers (Jansson‐Boyd and Kobescak 2020). Older adults and those with dysphagia are prone to anorexia and consequently malnutrition due to the unattractive appearance and texture of food (Pereira et al. 2021). Research has found that older adults with reduced appetite prefer food with color variations, high fiber, and firm texture (van der Meij et al. 2015). In addition, older adults show a preference for vanilla and lemon‐flavored ice cream as well as Swedish cheesecake, which is mainly attributed to the vibrant appearance and light, moist texture of these three food items (Wendin et al. 2021). There are also studies that have used 3D technology to transform the unappealing and difficult‐to‐swallow texture of wood ear pudding into visually appealing food, suitable for consumption by older adults with swallowing difficulties or related issues (Xing et al. 2022). This indicates that improving the appearance and texture of food can effectively increase older adults' acceptance and intake of food, thereby improving their nutritional status and quality of life.

## 5 Conclusions and Future Perspectives

### 5.1 Conclusions

This review comprehensively examines sensory decline in older adults and its impact on their dietary behavior, thoroughly analyzes the limitations of existing sensory evaluation methods in the elderly population, and summarizes various sensory compensation strategies that can enhance older adults' food preferences and intake. The study highlights that aging is associated with a general decline in sensory functions such as vision, hearing, olfaction, and taste, which can lead to reduced food perception, selection, and consumption, potentially resulting in malnutrition (Cavazzana et al. 2018; Doets and Kremer 2016). The exacerbation of sensory decline due to COVID‐19 further complicates dietary management in older adults (Burges Watson et al. 2021; Chaaban et al. 2021). While most sensory evaluation methods are designed for healthy adults, some can be adapted for use with older adults after modifications. For example, the TI method can assess dynamic sensory attributes like viscosity or flavor release (Hutchings et al. 2014; Luckett et al. 2016), and rapid methods like sorting and PM are accessible to older adults due to their simplicity (Scott et al. 2017; Mena et al. 2020). Additionally, the CATA method and JAR scale simplify the evaluation process, making them suitable for assessing older adults' food acceptance and preferences (Ares et al. 2014; Rutkowska et al. 2022). However, individual differences in cognitive function and sensory sensitivity among older adults must be considered when applying these methods. The review also presents a range of compensation strategies, including enhancing individual sensory attributes like sweetness and aroma, as well as multisensory approaches that improve food appearance, texture, and sound, thereby increasing older adults' food preferences and intake (Song et al. 2016; Thomas et al. 2021). These strategies offer promising solutions to mitigate the dietary challenges posed by sensory decline in older adults, contributing to improved nutritional status and quality of life.

### 5.2 Future Perspectives

To effectively address the challenges posed by sensory decline in older adults and improve their food preferences and nutritional intake, future research should focus on several strategic areas. First and foremost, it is crucial to deepen the understanding of the neurobiological mechanisms behind sensory decline. This includes investigating how these changes affect dietary behavior and nutritional intake through neurobiological pathways; examining individual differences, such as genetic factors, lifestyle, and chronic diseases, which play a significant role in modulating sensory decline, providing a solid foundation for developing personalized prevention and intervention strategies (Reuter‐Loryea et al. 2020; Wang et al. 2020); and additionally, continuing research on the long‐term impact of the pandemic on sensory functions, particularly the effects of neuroinflammation and damage to olfaction and taste (Iebba et al. 2021; Srinivasan et al. 2022). Second, developing innovative sensory evaluation methods for older adults, especially those in poor health, is essential. Leveraging information technology and neurobiological tools, such as EEG and FaceReader, can provide deeper insights into sensory processing (Poldrack and Wagner 2004). Lastly, a comprehensive exploration of sensory compensation strategies is necessary. The implementation of these strategies should be continuously optimized to improve their effectiveness. This includes developing personalized plans for older adults with varying health conditions, dietary habits, and cultural backgrounds (Wang et al. 2019). Long‐term studies are crucial for assessing the sustained impact of these strategies on health and nutritional status, ensuring their safety and sustainability (Regan et al. 2019, 2021). Interdisciplinary collaboration will further enhance the scientific validity and effectiveness of these strategies by integrating insights from psychology, nutrition, and food science (Fischer et al. 2009; Oleszkiewicz et al. 2020). Through ongoing research and innovation, we can provide older adults with more effective sensory compensation methods, improving their dietary health and overall quality of life.

## Author Contributions

**Yilin Li:** conceptualization (lead), formal analysis (lead), visualization (lead), writing – original draft (lead). **Shuying Wang:** manuscript preparation, data acquisition and analysis. **Lanxin Zhang:** data curation (equal), formal analysis (equal). **Qianhui Dong:** data curation (equal), formal analysis (equal). **Xinyu Hu:** data curation (equal), formal analysis (equal). **Yuxin Yang:** data curation (equal), formal analysis (equal). **Ting Liu:** conceptualization (equal), resources. **Baopei Wu:** writing – review and editing. **Bingqi Shan:** writing – review and editing. **Chuncao Yin:** writing – review and editing (equal). **Qinggang Xie:** conceptualization (equal). **Baoqing Zhu:** writing – review and editing, supervision, resources, project administration, conceptualization (equal). **Chengdong Zheng:** conceptualization (equal), project administration (equal).

## Conflicts of Interest

Yilin Li, Ting Liu, Bingqi Shan, Qinggang Xie, and Chengdong Zheng are employed by Feihe Dairy Co. Ltd., Tsitsihar, China. The other authors declare that they have no known competing financial interests or personal relationships that could have appeared to influence the work reported in this paper.

## Data Availability

Data will be made available upon request.
